# Supplementary material for: Chemical composition, antioxidant and antitumor activities of sub-fractions of wild and cultivated Pleurotus ferulae ethanol extracts
Source: PeerJ. 2018 Dec 20;6:e6097. doi: 10.7717/peerj.6097 (PMC6304266; doi:10.7717/peerj.6097)
Supplement: Supplemental Information 2 [file peerj-06-6097-s002.docx]

**Draft data**

For Fig. 3B, HeLa cells were treated with PFEE-W/C and sub-fractions and stained with hoechst 33342, then the morphology of nuclei was observed by inverted fluorescence microscopy.

Untreated
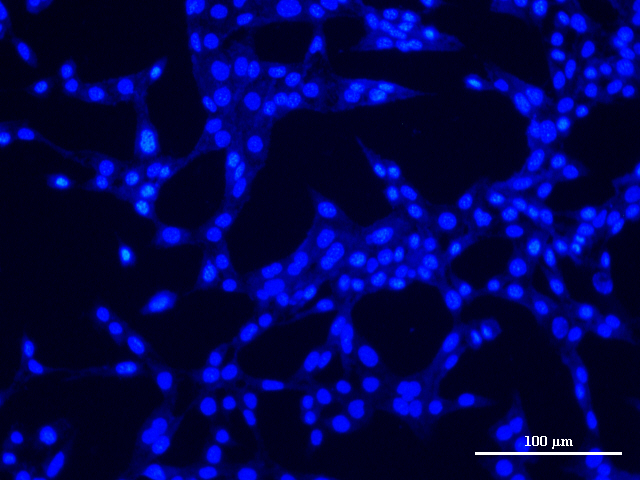


DMSO


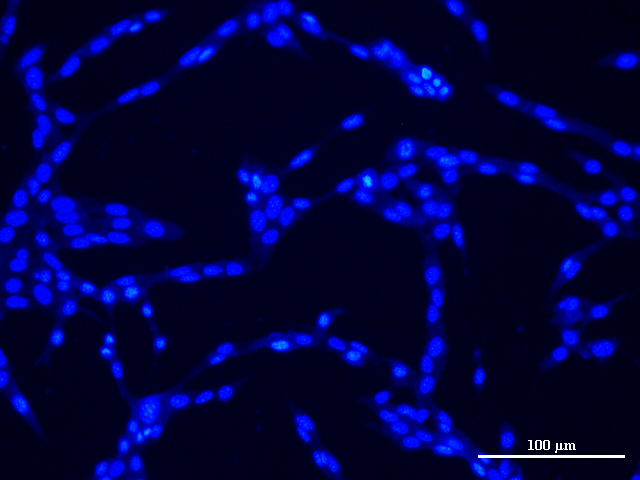


Cisplatin
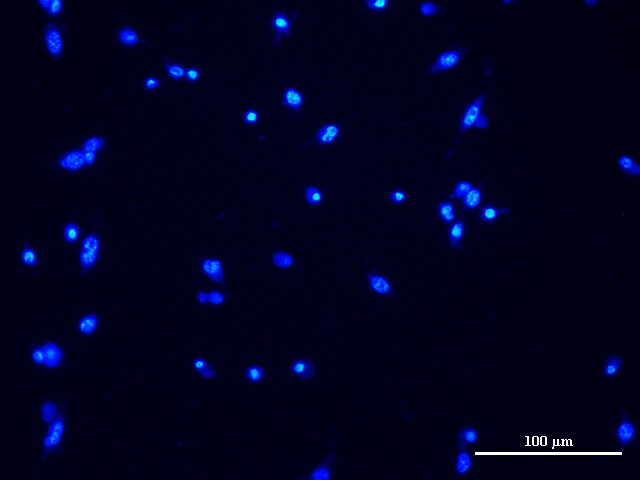


PFEE-W


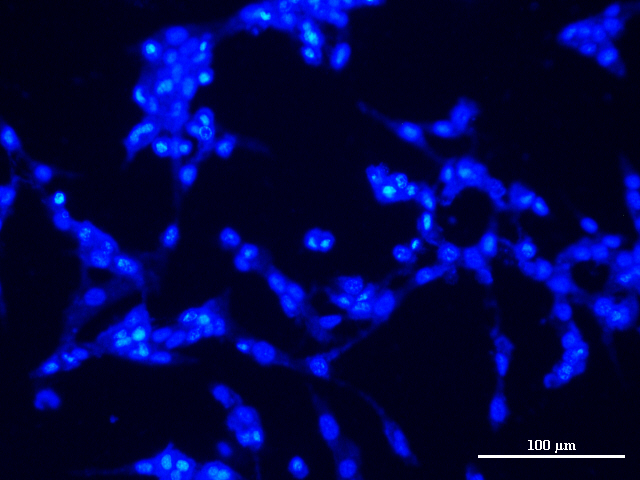


Pe-W
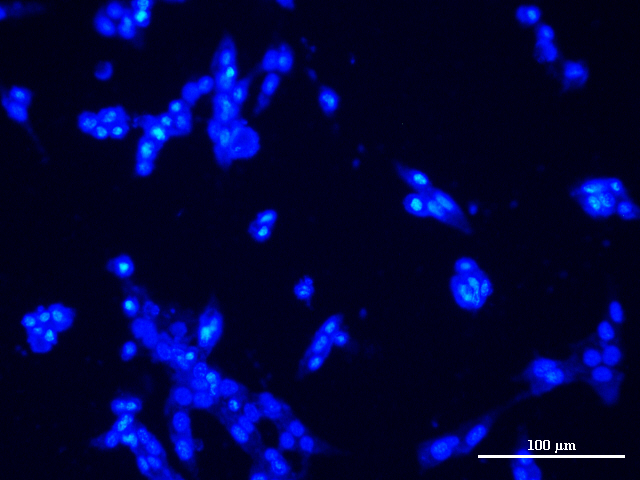


Ea-W
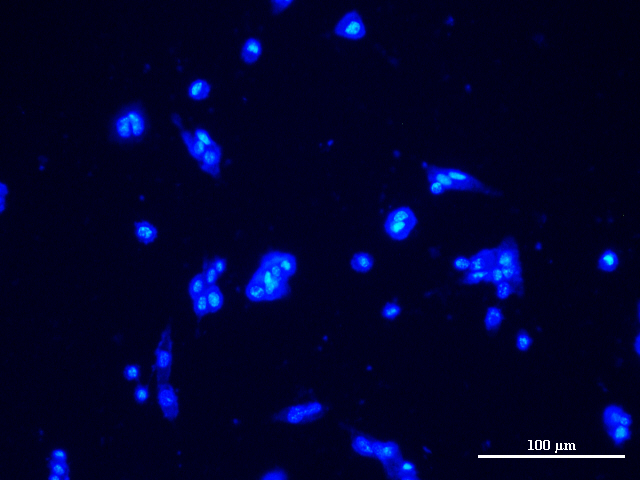


PFEE-C
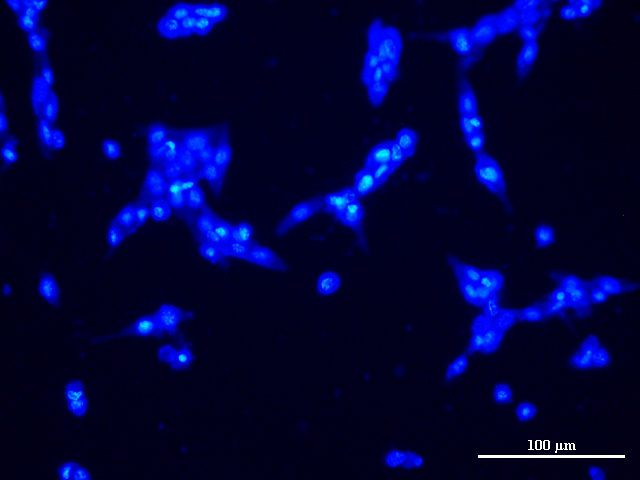


Pe-C
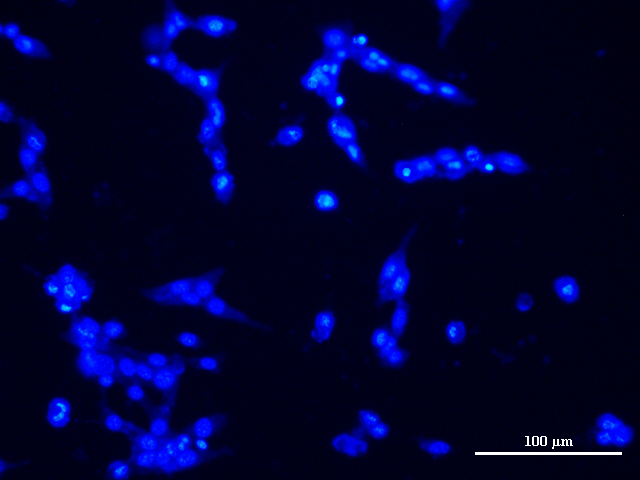


Ea-C


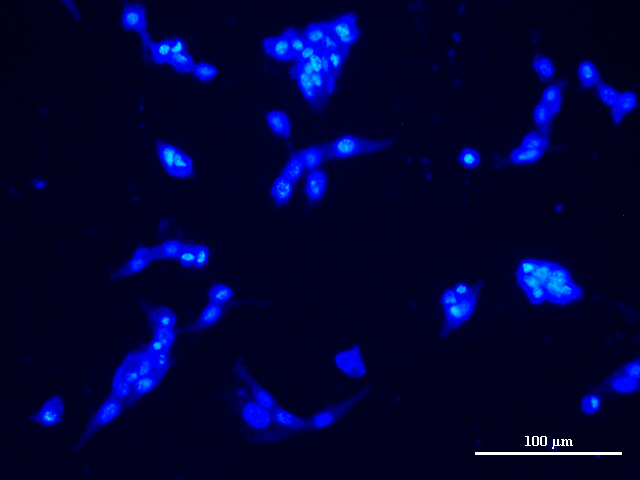


For Table 2 and 3, the chemical composition of fatty acids of Pe-W and Pe-C were detected by GC-MS. The total ion current chromatograms of fatty acids of Pe-W and Pe-C were shown.

Pe-W

Pe-C
