# Supplementary figures and images for: Chemical composition, antioxidant and antitumor activities of sub-fractions of wild and cultivated Pleurotus ferulae ethanol extracts
Source: PeerJ. 2018 Dec 20;6:e6097. doi: 10.7717/peerj.6097 (PMC6304266; doi:10.7717/peerj.6097)

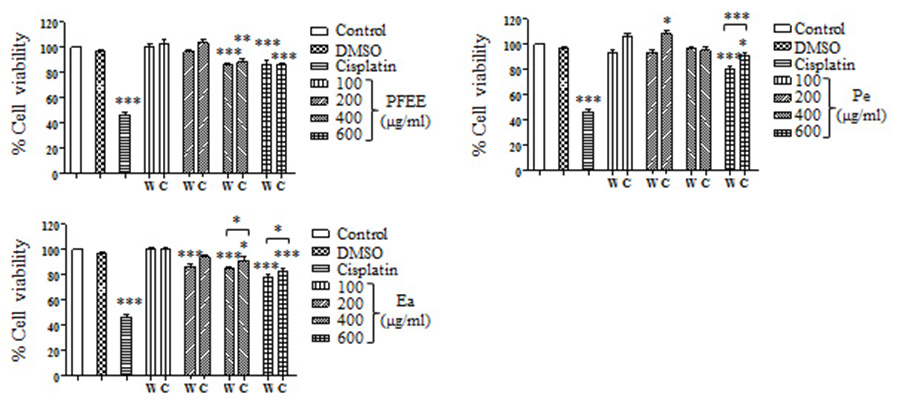

Supplement: Supplemental Information 3 — The viability of NCTC1469 cells after treatment with PFEE-W/C and their subfractions for 24 h. Data are analyzed by ANOVA. *p < 0.05; **p < 0.01; ***p < 0.001 compared to untreated group. The two-tailed paired t-test was used to compare wild and cultivated P. ferulae extracts. *p < 0.05; ***p < 0.001. [file peerj-06-6097-s003.jpg]

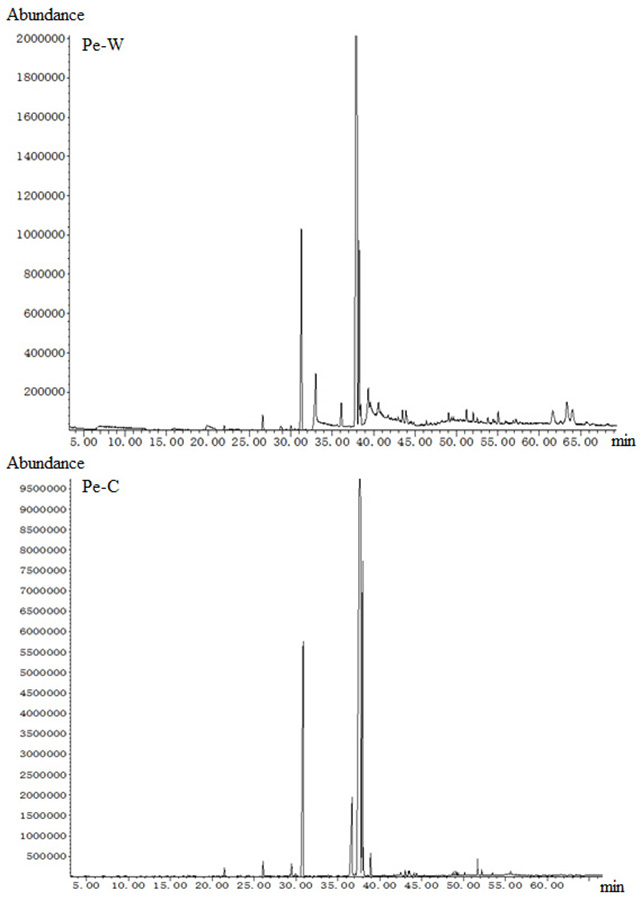

Supplement: Supplemental Information 4 [file peerj-06-6097-s004.jpg]
